# Potential Neuroprotective Effects of Docosahexaenoic Acid on Glutamate-Induced Neurotoxicity: A Systematic Review of Pre-Clinical Studies

**DOI:** 10.3390/nu18111819

**Published:** 2026-06-04

**Authors:** Muhammad Hani Rahimi Rusleen, Nur Izzati Mansor, Adila A. Hamid, Nurul Hafizah Mohd Nor, Zainah Mohamed

**Affiliations:** 1 Department of Nursing, Faculty of Medicine, Universiti Kebangsaan Malaysia, Cheras 56000, Kuala Lumpur, Malaysia; mhrahimi1301@gmail.com (M.H.R.R.); zainah_mohamed@ukm.edu.my (Z.M.); 2 Department of Physiology, Faculty of Medicine, Universiti Kebangsaan Malaysia, Cheras 56000, Kuala Lumpur, Malaysia; adilahamid@ukm.edu.my; 3 Institute of Islamic Civilization, Universiti Kebangsaan Malaysia, Bangi 43600, Selangor Darul Ehsan, Malaysia; nurulhafizah@ukm.edu.my

**Keywords:** docosahexaenoic acid, glutamate, excitotoxicity, neuroprotection

## Abstract

**Background/Objectives:** Excitotoxicity, primarily caused by excessive glutamate signaling, is a significant contributor to the aetiology of several neurological disorders. Docosahexaenoic acid (DHA), a long-chain omega-3 polyunsaturated fatty acid, is known for its neuroprotective properties, including antioxidants and anti-inflammatory effects. However, the existing literature has not sufficiently reviewed its specific role in glutamate-induced excitotoxicity. This systematic review aimed to provide comprehensive information from the literature on the neuroprotective effects of DHA in models of glutamate-induced neurotoxicity. **Methods:** A systematic search was conducted in the Cochrane Library, Scopus, Web of Science, PubMed, ScienceDirect, and Google Scholar, following PRISMA 2020 guidelines. The following keywords were used: DHA OR docosahexaenoic acid AND excitotoxicity OR glutamate-induced excitotoxicity OR glutamate-induced neurotoxicity. A total of 475 articles were screened, and 13 original articles published between 2003 and 2025 were included for data extraction. These studies included nine in vivo animal studies, three ex vivo studies, and one in vitro study. The risk of bias was assessed using SYRCLE’s methodology. **Results:** Our findings demonstrate that DHA provides substantial neuroprotection against excitotoxicity through antioxidative, anti-inflammatory, and anti-apoptotic mechanisms. Furthermore, DHA enhances neuronal function and cognitive performance by modulating neurotransmitter levels and glutamate-related signaling pathways. Despite these positive outcomes, heterogeneity across studies suggests that the neuroprotective properties of DHA may be affected by various parameters, such as the source of DHA, treatment dose and duration, age and experimental design. **Conclusions:** Although previous studies have demonstrated the benefits of DHA in preclinical and clinical settings of neurological disorders, further clinical studies focusing on the modulation of excitotoxicity by DHA are needed to validate its translational efficacy and therapeutic significance.

## 1. Introduction

Glutamate is the major excitatory neurotransmitter in the mammalian central nervous system (CNS), responsible for synaptic transmission. It also plays a key role in synaptic plasticity, a cellular mechanism underlying cognition and memory formation [1,2], as well as synaptogenesis [3,4]. Glutamate exerts its excitatory effects by activating its primary ionotropic receptors, *N*-methyl-D-aspartate (NMDA) and α-amino-3-hydroxy-5-methyl-4-isoxazolepropionic acid (AMPA) receptors, which are found on postsynaptic neurons. Activation of AMPA receptors facilitates sodium (Na^+^) influx and rapid postsynaptic depolarization, eliminating the magnesium (Mg^2+^) block and activating NMDA receptors. Activation of NMDA receptors initiates a calcium (Ca^2+^)-dependent signaling pathway, leading to synaptogenesis and synaptic remodeling required for learning and memory [5,6,7,8,9]. Upon receptor activation, glutamate is rapidly cleared from the synaptic cleft to maintain extracellular homeostasis, primarily by astrocytes via excitatory amino acid transporters (EAATs). In astrocytes, glutamate is converted into glutamine by glutamine synthetase for recycling. The synthesized glutamine is transported to neurons, converted into glutamate by glutaminase, and stored in synaptic vesicles at presynaptic terminals, thus contributing to the glutamate–glutamine cycle [10,11,12,13].

The disturbances in the glutamatergic system may disrupt normal physiological functions, leading to excitotoxic neuronal damage and death [14,15]. Glutamate excitotoxicity is a condition characterized by abnormally high glutamate levels in the brain’s extracellular space due to an imbalance between glutamate release and clearance, resulting in glutamate receptor overactivation. A high level of glutamate leads to continuous binding of glutamate to the receptors that disrupts Ca^2+^ homeostasis, causes mitochondrial dysfunction, induces oxidative stress, activates the apoptotic pathway, and ultimately leads to neuronal death [16,17,18]. Glutamate excitotoxicity has been implicated in several neurological disorders, including Alzheimer’s disease (AD) [19,20,21], Parkinson’s disease (PD) [22,23,24], traumatic brain injury (TBI) [10,25,26], and ischaemic stroke [27,28,29]. Importantly, glutamate excitotoxicity often arises as a secondary consequence of pathological changes associated with these disorders, leading to glutamate dysregulation and exacerbating neuronal damage. For example, in AD, the aggregation of amyloid beta (Aβ) peptides may induce the release of D-serine, an NMDA receptor co-agonist, and trigger the release of glutamate [30] through activation of *N*-type voltage-gated Ca^2+^ channels [31], ultimately contributing to excitotoxic events. Whilst in PD, overexpression of alpha (α)-synuclein increases phosphorylation of NMDAR subunits (NR1 and NR2B) in the substantia nigra and striatum, thereby increasing vulnerability to glutamate excitotoxicity [32]. Furthermore, iron-induced α-synuclein has been reported to enhance synaptic transmission via AMPA receptor activation, resulting in excitotoxic neuronal death [24]. In TBI, the breakdown of the blood–brain barrier and neuronal death lead to excessive and unregulated release of glutamate [33]. Moreover, the cellular expression of EAAT1 and EAAT2 is diminished following TBI, hence suppressing glutamate uptake by astrocytes [34]. During ischaemia, reduced cerebral blood flow causes glucose and oxygen deprivation. This disrupts mitochondrial oxidative phosphorylation, resulting in adenosine triphosphate (ATP) depletion. Low ATP levels lead to failure of the Na^+^/K^+^ ATPase pump, causing neuronal membrane depolarization and uncontrolled glutamate release and uptake. Excess extracellular glutamate may contribute to intracellular Ca^2+^ overload, subsequently initiating excitotoxic cascades [28,29]. Thus, modulating the glutamate signaling pathway offers a potential therapeutic strategy for neuroprotection.

**Figure 1:**
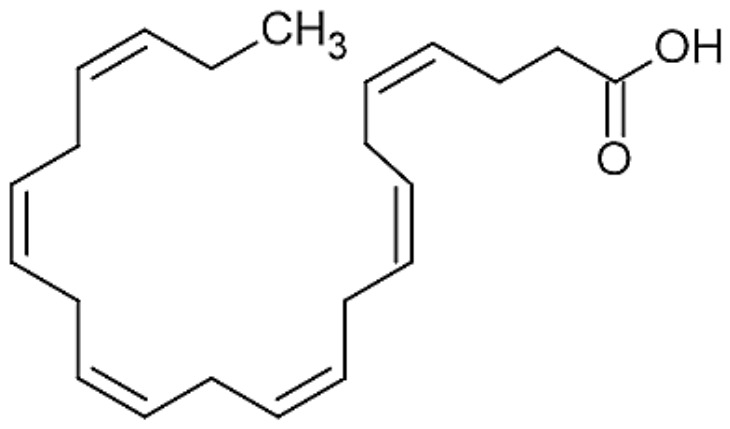
Chemical structure of docosahexaenoic acid (DHA).

Dietary bioactive compounds have emerged as promising therapeutic candidates for attenuating excitotoxic neuronal damage due to their multiple bioactivities, including antioxidant, anti-inflammatory, and anti-apoptotic effects, which enable them to modulate several interrelated pathways involved in excitotoxicity. One such bioactive compound is docosahexaenoic acid (DHA), a long-chain polyunsaturated fatty acid (LC-PUFA) of the omega-3 family, featuring a 22-carbon chain and six cis double bonds (Figure 1) [35]. DHA is highly abundant in membrane phospholipids in the mammalian brain, particularly in gray matter, and is also present in the retina [36,37]. Remarkably, this endogenous DHA plays a crucial role in mediating neurological functions by modulating neurotransmission, synaptic plasticity, neurogenesis and synaptogenesis, neuroinflammation, as well as synaptic integrity [38,39,40,41,42,43,44,45]. Furthermore, DHA is considered essential for fetal brain development and early infancy [46,47,48].

The brain obtains DHA either by endogenous synthesis from α-linolenic acid (ALA) via the desaturation–chain elongation pathway in the liver or through dietary intake of DHA. However, the liver’s capacity to synthesize DHA is lower in humans [35,43,49,50]. Dietary DHA is mainly obtained from marine species such as fatty fish, shellfish, and microalgae [35,51,52]. Interestingly, the bioavailability of DHA from existing supplements is inadequate because the adult brain has limited absorption capability, which remains a critical factor affecting its physiological effects. Recent studies have shown that esterifying DHA to lysophosphatidylcholine (LPC) effectively enhances brain DHA levels compared to DHA-triacylglycerol (TAG) and di-DHA phosphatidylcholine (PC) [53]. Dietary DHA has been extensively studied for its neuroprotective effects in various pathological conditions, owing to its antioxidant and anti-inflammatory properties [54,55,56].

Several studies have shown that DHA supplementation positively affects glutamatergic synaptic activity by regulating glutamate homeostasis. For example, a deficiency of dietary *n*-3 fatty acids in adolescent rats resulting in decreased DHA levels in the frontal cortex and significant increases in glutamate levels. In contrast, dietary fish oil supplementation increases brain DHA levels and decreases glutamate levels [57]. Moreover, DHA deficits in elderly brains reduce the levels of synaptic proteins such as AMPA2, NR2, and PSD-95, which are essential for glutamatergic transmission [58]. Furthermore, deficiency of *n*-3 fatty acids during development led to reductions in synapsin and glutamate receptor subunit levels in the hippocampus, resulting in impaired long-term potentiation (LTP), a mechanism underlying learning and memory [45]. In addition, DHA may affect the glutamatergic system by modulating EAAT2 activity and maintaining glutamate uptake [59]. These findings elucidate the extensive roles of DHA in the brain’s regulation and maintenance of glutamatergic signal transmission.

While several recent comprehensive reviews have examined the neuroprotective properties of DHA in healthy individuals and against neurological disorders [60,61,62,63], a systematic comparison specifically addressing its role in modulating glutamate-induced excitotoxic pathways has not yet been conducted. This review, therefore, aims to systematically evaluate the potential neuroprotective effects of docosahexaenoic acid on glutamate-induced neurotoxicity. The review focuses on pre-clinical studies to minimize confounding variables commonly present in human clinical research, such as socioeconomic factors, medication use, and pre-existing co-morbid and psychiatric conditions. Preclinical studies also allow precise control over treatment dosage, timing, and duration, as well as direct access to brain tissues, offering a better understanding of DHA’s direct neurobiological effects on glutamate signaling.

## 2. Materials and Methods

### 2.1. Search Strategy

The systematic review was conducted in accordance with the Preferred Reporting Items for Systematic Reviews and Meta-Analyses (PRISMA) Guidelines [64]. The protocol of this study is available on the INPLASY website under the registration number INPLASY202650047 (https://inplasy.com/inplasy-2026-5-0047/, accessed on 8 May 2026) [65]. Relevant literature was identified from five primary databases: Cochrane Library, Scopus, Web of Science, PubMed, and ScienceDirect. The following keywords were used: (“DHA” OR “docosahexaenoic acid”) AND (Excitotoxicity OR “Glutamate-induced Excitotoxicity” OR “Glutamate-induced neurotoxicity”). A similar search was also conducted using Google Scholar. Harzing’s Publish or Perish version 8 software was used to retrieve the most relevant studies. Publication date and country were not restricted.

### 2.2. Eligibility and Exclusion Criteria

This review included full-length original research articles published in the English language. All pre-clinical studies (in vivo, ex vivo, and in vitro) investigating neuroprotective effects of DHA against glutamate-induced neurotoxicity or excitotoxicity models, with consideration given to various routes of administration, dosages, and treatment durations, were included. Combination dosing of DHA and EPA, such as in fish oil, was included only if the DHA concentration was specified. Neuronal cultures (in vitro or ex vivo) or animal models exposed to glutamate excitotoxicity, including those using glutamate receptor agonists or any CNS-related models directly assessing glutamate dysregulation or excitotoxicity, were included.

However, all studies that examined only the relationship between DHA supplementation and bioavailability, or an increase in brain DHA, were excluded unless they also assessed neuroprotection related to glutamate excitotoxicity. Studies describing neuroprotection without the use of DHA and studies using DHA but not employing an excitotoxicity model were also excluded. Review articles, news articles, book chapters, conference proceedings, editorial letters, and case studies were also excluded from this review.

### 2.3. Study Selection and Data Extraction

The studies included in this review were screened according to the PICOS framework, as follows:

(1) Population (P): Neuronal cultures (in vitro or ex vivo) or animal models exposed to glutamate excitotoxicity (including glutamate receptor agonists or any CNS-related models that directly assess glutamate dysregulation or excitotoxicity) were included;
(2) Intervention (I): Studies using DHA as an intervention in the experimental group were included;
(3) Comparison (C): Comparator groups received either no intervention or were treated with a relevant conventional drug or fatty acid;
(4) Outcome (O): Changes in cell viability, morphology, apoptosis, oxidative stress, neuronal or synaptic markers, glutamate receptor or transmission regulation, or any relevant neurobehavioral outcomes;
(5) Study design (S): Pre-clinical (in vitro, in vivo, and ex vivo) studies.

Initially, articles were screened by one reviewer (MHR) based on article type, title, language, and abstract related to the effect of DHA on glutamate-induced neurotoxicity. Duplicates were removed. In the second screening, studies were screened by two reviewers (MHR and NIM) based on inclusion and exclusion criteria. Articles not meeting the inclusion criteria were excluded. Any similar studies were removed. Subsequently, the full texts of potentially eligible studies were retrieved and independently assessed for eligibility by two reviewers (MHR and NIM). Any disagreements were resolved through discussion with a third reviewer (NHMN).

The study characteristics extracted from the eligible full-text articles included the following categories: (i) bibliographic details (title, authors, publication year, journal); (ii) study design (animal or neuronal cultures representing excitotoxicity model, experimental types); (iii) DHA used (source, dosage, duration of treatment); (iv) outcomes (cell viability, morphology, apoptotic marker expression, glutamate levels, oxidative stress status, inflammatory response, glutamate receptor activity, neuronal marker expression, dendritic integrity and cognitive function). For studies with multiple interventional arms, only data from relevant arms were considered.

### 2.4. Risk of Bias Assessment

Two reviewers (MHR and NIM) independently assessed the methodological studies using the Systematic Review Centre for Laboratory Animal Experimentation (SYRCLE) tool for in vivo studies [66]. The main components of this assessment were: (1) selection bias: random sequence generation, baseline characteristics, allocation concealment; (2) detection bias: random housing, blinding, random outcome assessment; (3) attrition bias: incomplete outcome data; (4) reporting bias: selective reporting; and (5) other bias. For in vitro studies, a customized Joanna Briggs Institute (JBI) checklist for non-randomized experimental studies was used, comprising: (1) reporting bias: source of DHA, cell origin, duration of treatment and DHA concentration; (2) performance bias: positive or negative control, blinded investigator, reliable tool, and random assessor; (3) detection bias: triplicate, more than one independent experiment, outcome assessor blinded; (4) selection bias: missing data reported [67]. Each domain was labeled as “Yes” for low risk of bias, “Unclear” for some risk of bias, and “No” for high risk of bias. Any disagreements were resolved through discussion with a third reviewer (NHMN).

## 3. Results

### 3.1. Studies Selected

**Figure 2:**
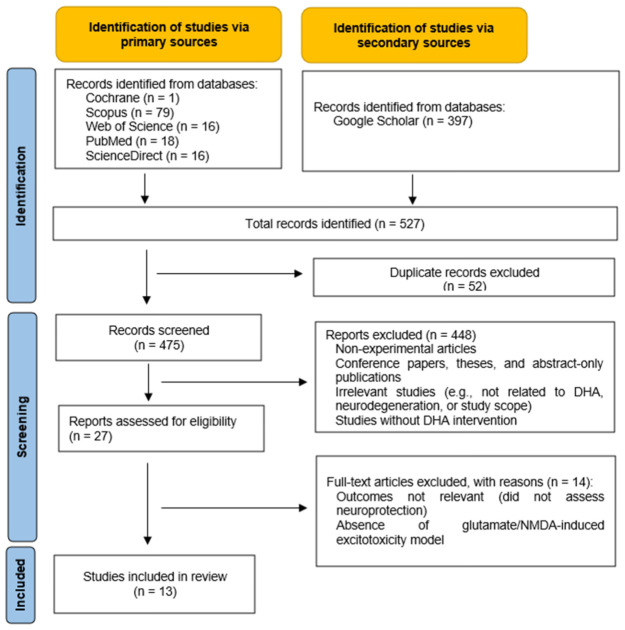
Preferred Reporting Items for Systematic Review and Meta-Analyses (PRISMA) 2020 flow diagram for systematic review.

Throughout the article selection process, a total of 527 records were identified from primary sources (Cochrane, Scopus, Web of Science, PubMed, and ScienceDirect) and a secondary source (Google Scholar). After title screening, 52 duplicate articles were removed. A total of 475 articles were then assessed based on their abstracts according to the inclusion criteria, resulting in the exclusion of 448 studies. The remaining 27 full-text articles were assessed for eligibility, and 14 studies were excluded with reasons provided. Finally, 13 studies published between the years 2003 and 2025, all of which met the inclusion criteria, were included in this review and assessed for risk of bias. The following information was extracted from the studies: study design, DHA source, parameters investigated, analysis technique, results, and conclusions. The selection process is outlined in Figure 2.

### 3.2. Risk of Bias

**Figure 3:**
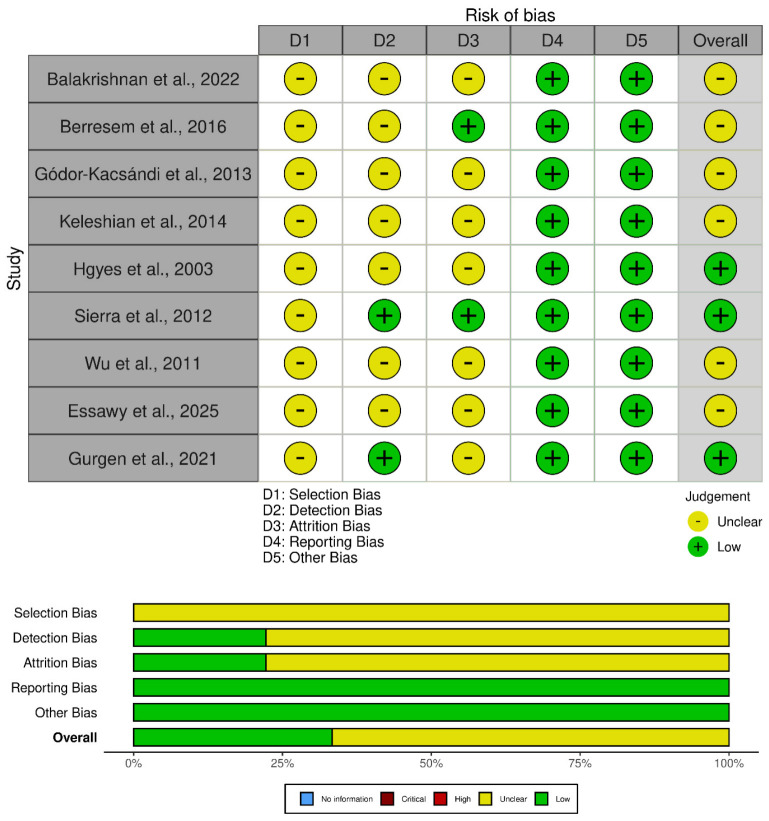
Systematic Review Centre for Laboratory Animal Experimentation (SYRCLE) risk of bias summary: review authors’ assessments of each risk of bias item for each included study [68,69,70,71,72,73,74,75,76].

**Figure 4:**
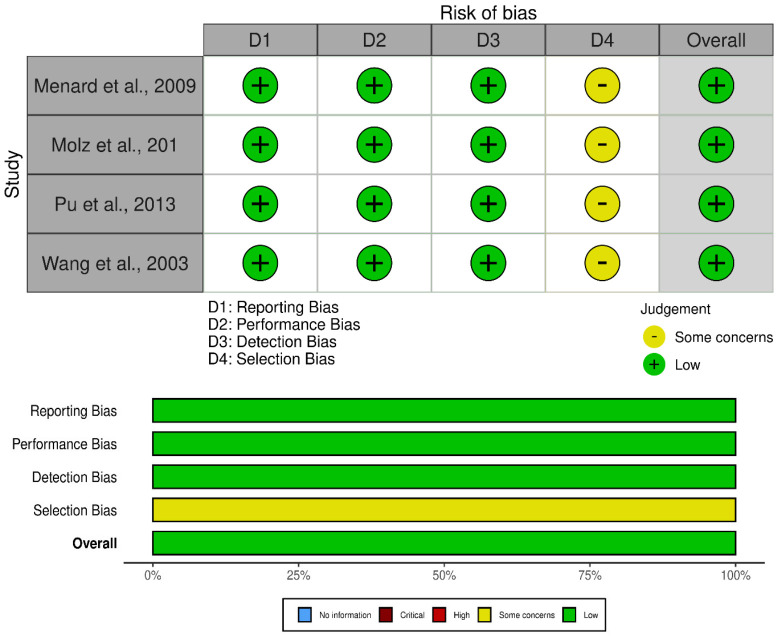
The Joanna Briggs Institute (JBI) critical evaluation checklist summary is used to assess in vitro and ex vivo Studies [56,77,78,79].

The risk of bias assessment for the in vivo studies and for the in vitro and ex vivo studies is presented in Figure 3 and Figure 4, respectively. Of the 13 publications, six studies were categorized as having some concerns, while the remaining seven had a low risk of bias. Most bias was related to selection bias, due to issues identified in sample randomisation before the start of experiments. Publications assessed using SYRCLE indicated that more than 50% had some concerns regarding selection bias, detection bias, and attrition bias. Publications assessed with the customized JBI tool had some concerns regarding selection bias. Nevertheless, all publications included in this study had a very low risk of reporting bias, indicating transparency in the data included and analyzed in their studies.

### 3.3. Study Characteristics

The summary of the characteristics of the selected studies is described in Table 1 and Table 2. All studies were pre-clinical; nine studies involved in vivo models, three studies involved ex vivo models, and one study involved an in vitro model. In in vivo studies, most publications used rodent models, while only Sierra et al. [68] used zebrafish to recreate the excitotoxicity model. Four studies involved maternal DHA supplementation during pregnancy [68,69,70,71], with supplementation continuing until the birth of the first offspring, while the remaining studies were tested on the adult animal. For ex vivo studies, three publications utilized hippocampal slices from a rat model [56,77,78], but only Pu et al. [79] used primary cultures of oligodendrocytes for DHA pretreatment prior to exposure to excitotoxic insults. Excitotoxicity models were created using various excitotoxic chemicals: four studies used toxic NMDA [70,71,72,77], two studies used AMPA [77,79], two studies used Kainate Acid (KA) [68,77], and five studies used research-grade glutamate in the form of monosodium glutamate or L-glutamate purchased from accredited suppliers [56,69,72,75,78]. Indirect excitotoxicity models were also introduced in two publications: in a study by Wu et al. [76], the rats underwent Fluid Percussion Injury (FPI) to represent traumatic brain injuries, and Berressem et al. [73], which used the MCAO model to mimic ischaemic stroke. Both types of neurodegenerative models are further affected by dysregulation of the glutamate neurotransmitter [10,80].

DHA is a naturally occurring substance and can be sourced from either fish oil or DHA-enriched microalgae. In this review, six studies sourced their DHA from fish oil [69,70,71,72,73,74], while only one study used microalgal-based DHA to compare with fish oil-based DHA [69]. Two studies did not explicitly report the source of DHA used in the experiments [68,75]. Furthermore, in the selected study, the tested dose of DHA ranged from as low as 1.4 mg/kg BW up to 300 mg/kg BW in experiments involving in vivo models. Concentrations of DHA were used in cellular models, with the lowest being 5 µM [56] in the culture media and the highest being 90 µM [77]. DHA concentrations were tested for lethal dose prior to testing against excitotoxicity.

The route of administration was also considered, as some methods of DHA administration may yield better results. The most common route in in vivo models was dietary supplementation [68,69,70,71,76], while 2 out of 9 studies used intraperitoneal injection [42,47], and the others used oral gavage [74,75]. In contrast, in vitro models used only culture media to pre-treat brain cells with DHA [56,77,78,79].

### 3.4. Effects of DHA on Neuronal Cell Survival and Apoptosis

The effects of DHA on the viability and survival of neuronal cells in both physiological and pathological conditions have been extensively studied. DHA supplementation has been shown to prevent further neuronal death by protecting cells against excitotoxicity. This review included five in vivo, as well as four in vitro and ex vivo studies reporting the effects of DHA on neuronal survival and apoptosis.

For example, Hgyes et al. [71] showed that maternal dietary fish oil (Marinol D40) supplementation containing 13.6% DHA per 100 g of diet attenuated excitotoxic neuronal damage by reducing cholinergic neuronal loss in NBM, with less than 20% cell loss observed, and preserved dendritic and axonal integrity in the offspring following 48 h of NMDA-induced excitotoxicity. Balakrishnan et al. [69] found that maternal DHA supplementation (1.4 and 2.9 mg/kg BW/day), followed by a 30-day treatment in the offspring, significantly reduced MSG-induced neuronal damage and apoptotic features in the offspring’s cerebrum.

In line with these findings, DHA was found to increase CREB and BDNF mRNA expression in offspring from DHA-supplemented mothers, suggesting activation of neuronal survival mechanisms [69]. Similarly, in the FPI-induced TBI rat model, Wu et al. [76] found that a DHA-enriched diet promoted neuronal survival by restoring CREB and BDNF levels in the hippocampus. Another study by Gürgen et al. [75] (2021) reported that oral administration of DHA (300 mg/kg BW) for nine days increased BDNF protein expression in the hippocampal region, suggesting enhanced neuronal survival against MSG-induced excitotoxic damage.

Essawy et al. [74] reported that oral treatment with ω−3 PUFAs (48 mg/kg) for four weeks significantly improved histopathological changes in the cortical area of MSG-treated rats. Furthermore, Berressem et al. [73] reported that intravenous administration of OGV 10%^®^ (~90 mg/kg BW DHA) reduced the infarcted area by 56% and stroke severity by 60% in MCAO mice, suggesting its contribution to neuroprotective effects.

These findings are further supported by in vitro and ex vivo studies, which demonstrate that DHA has comparable cellular protective properties. Three ex vivo studies using hippocampus slices showed improved neuronal survival following DHA pre-treatment. These studies found that DHA restored cell viability and suggested a protective effect under excitotoxic conditions [56,77,78]. The protective effect of DHA is not restricted to neurons. DHA has also been shown to enhance the survival of oligodendrocyte cells exposed to AMPA-induced excitotoxicity [49].

In addition to promoting neuronal survival in excitotoxicity, DHA was found to modulate apoptotic signaling pathways. Two studies reported that DHA attenuated apoptosis in MSG-induced excitotoxicity in adult [74] and young rats from DHA-supplemented mothers [69], by regulating apoptotic activity such as caspase-3 and Bcl-2 [69].

### 3.5. Effects of DHA on Glutamate Signaling

Glutamate-mediated excitotoxicity is primarily caused by excessive stimulation of glutamate receptors, particularly NMDA and AMPA receptors, resulting in increased intracellular Ca^2+^ influx and subsequent neuronal damage and death. The effects of DHA-mediated glutamate receptor regulation vary between studies. For example, Balakrishnan et al. [69] and Menard et al. [77] observed that DHA treatment reduced the expression of NMDA and AMPA receptors, respectively, suggesting a potential role of DHA in modulating glutamate receptors in excitotoxic conditions. In contrast, Gürgen et al. [75] reported that MSG exposure decreased NMDA receptor expression, which was then restored following DHA supplementation.

Beyond receptor-level modulation, DHA may exert its effects by modulating downstream synaptic plasticity associated with glutamate transmission. For example, Wu et al. [76] found that DHA supplementation restored the expression of CaMKII and Synapsin I in FPI-induced TBI rats, both of which are essential markers for learning and memory processes.

### 3.6. Oxidative Stress Reduction and Anti-Inflammatory Effects of DHA

DHA supplementation has been shown to reduce excitotoxicity-induced oxidative stress associated with neuronal damage, as evidenced by reduced lipid peroxidation markers such as MDA in in vivo models and decreased TBAR levels in ex vivo systems, indicating reduced ROS-mediated damage [56,74]. Consistent with these findings, Wu et al. [76] demonstrated reduced lipid peroxidation in FPI-induced TBI rats, indicated by lower 4-HNE levels. The levels of ROS, such as NO, were also regulated by DHA supplementation. An in vitro study by Wang et al. [78] indicated a reduction in the level of NO upon DHA pre-treatment, with a reduced calcium ion influx indicating the plausible role of DHA in regulating glutamate receptors. An in vivo study by Berressem et al. [73] found that the mitochondrial function was restored with higher levels of MMP, ATP production, and RCR upon DHA supplementation, supporting the role of DHA in maintaining the production of ROS in mitochondria after glutamate excitotoxicity.

In parallel, DHA regulates oxidative stress through enhancing endogenous antioxidant defense systems, with several investigations demonstrating elevated levels of antioxidant markers such as GSH, GPx, and CAT [69,74,76,78]. Moreover, Wu et al. [76] reported that dietary supplementation with DHA upregulates the antioxidant SOD and Sir2 protein levels. Sir2 promotes stress resistance through the activation of endogenous antioxidant activities and the maintenance of energy homeostasis. However, certain inconsistencies have been observed in SOD levels following DHA treatment. While Wang et al. [78] found that DHA treatment increased SOD levels, other reports indicate otherwise [69,74]. Overall, these findings indicate that DHA provides neuroprotective effects by alleviating oxidative stress and enhancing antioxidant activity under excitotoxic conditions.

Apart from its antioxidant properties, DHA has been found to modulate inflammatory responses associated with excitotoxic damage. In excitotoxicity, dysregulated glutamate signaling disrupts neuronal homeostasis and activates glial cells, including microglia and astrocytes, leading to the production of pro-inflammatory cytokines and mediators such as TNF-α, IL-1β, and IL-6. DHA has been suggested to modulate these inflammatory responses, thereby decreasing inflammation-induced neuronal damage. For instance, following DHA treatment, COX-2 levels were downregulated at stroke onset in a rat model [72,73]. Furthermore, reductions in IL-6, IL-1B, and TNF cytokines were observed in multiple excitotoxic models after DHA treatment [73,74].

### 3.7. Effects of DHA on Cognitive Function and Motor Recovery

Cognitive dysfunctions have been observed in multiple neurodegenerative diseases, with excitotoxicity becoming one of the causal pathways. In vivo studies have measured cognitive and motor recovery in excitotoxic animals treated with DHA. In a zebrafish model of epilepsy, a DHA-enriched diet reduced seizure activity, with the most significant effects observed in offspring of DHA-supplemented mothers [68]. This effect is further supported by another experimental model, including FPI-induced TBI rats. Wu et al. [76] found that DHA supplementation improved the rats’ learning performance in the Morris water maze, as indicated by shorter escape latency, suggesting improved spatial learning and memory. Despite its cognitive effects, DHA supplementation had no significant effect on motor performance; however, a reduction in neuronal lesions was observed [70].

## 4. Discussion

The potential of DHA as a treatment option for neurological disorders is widely acknowledged. However, its specific mechanism in the modulation of excitotoxicity remains incompletely elucidated. To our knowledge, this is the first systematic review to integrate preclinical findings on the effects of DHA supplementation in glutamate-mediated neurotoxicity models, with particular focus on how the source of DHA, age, treatment duration and dosage, and study design affect the research outcomes.

To summarize, the findings suggest that DHA exerts its neuroprotective effects against excitotoxic damage in various excitotoxicity models, including MSG-induced rats [69,74] and mice [75], L-glutamate-induced rat pups [76] and mice [56], FPI-induced TBI rat [52], KA-induced excitotoxicity in zebrafish [68] and rats [77], AMPA-induced rats [48], NMDA-induced rats [70,71,72,77], as well as MCAO mice [73], as demonstrated by reduced neuronal apoptosis and oxidative stress. The neuroprotective effects of DHA were also prominent in low to high dosage treatment. Nonetheless, higher doses of DHA supplementation increase the level of NMDA receptors, indicating its role in regulating glutamatergic transmission [69,75]. However, the heterogeneity of daily dosage may interfere with the reproducibility of the results, as the dosage used in each experiment might be dependent on types or organisms, disease induction, and route of administration (oral gavage, dietary supplements, or injections). Further research needs to be done to compare and validate the best methods and dosages for DHA treatment.

In addition, some concerns observed in the risk of bias assessment may also interfere with the final results. In vivo studies raised concerns about selection bias, indicating some bias with a lack of randomization at the start of the experiment. Assessments of the candidates were also detected to be influenced by bias when assessments were not randomized and the assessors were also not blinded. Attrition bias that is related to incomplete data reporting was also prominent in animal studies, which can reduce the data reliability, as the data reported in each experiment were inconsistent.

DHA modulates oxidative stress by regulating antioxidants. Oxidative stress, which is caused by the high level of ROS, can be reduced with DHA through the activation of antioxidative pathways. Studies by [55,81] indicated that DHA protects against H_2_O_2_ induction. Further in their research, antioxidative markers such as Nrf2 and NFE2L2 were found to increase after DHA pre-treatment and H_2_O_2_ induction. Downstream, DHA possesses the indirect endogenous antioxidant enhancement ability [82]. During excitotoxic conditions, DHA is metabolized to 4-hydroxy-2-hexenal (HHE), which upregulates the Nrf2 signaling pathway. Activation of Nrf2 stimulates the production of antioxidant enzymes, including glutathione peroxidase (GPx), enabling the detoxification of free radicals [83,84].

Additionally, DHA has considerable anti-inflammatory properties that help prevent neuronal death during excitotoxicity. Previous study reported that DHA may be metabolized to produce specialized pro-resolving mediators (SPMs) [85] such as resolvins, protectins, and maresins, all of which are synthesized through the oxidation of DHA by COX [66]. These mediators inhibit microglial activation and reduce pro-inflammatory cytokines, including TNF-α, IL-1β, and IL-6 [86]. In addition, Neuroprotectin D1 (NPD1), a potent anti-inflammatory and mediator derived from DHA, has been shown to prolong neuronal lifespan by modulating inflammatory signaling, mainly TNF-α and IL-6, and facilitating cellular repair pathways. In a previous study, NPD1 has been shown to protect neurons through upregulating Iduna expression in ischemic stroke [87]. Another study indicates that the injection of NPD1 facilitates the modulation of inflammation, particularly TNF-α and IL-6, in aging murine models [88]. This corresponds to the direct involvement of NPD1 in inhibiting macrophage infiltration in the region surrounding the injury, thus promoting resolution and repair [89]. Moreover, these anti-inflammatory properties complement DHA’s antioxidative activities, contributing to a neuroprotective state.

Preclinical studies indicate that DHA may influence several injury-related pathways, notably through its effects on synaptic function and glutamatergic transmission. However, translation to clinical settings must be approached with caution, as variations in pathology complexity, DHA dose, treatment duration, formulation, and patient characteristics may affect therapeutic outcomes. Thus, further clinical investigations are necessary to confirm whether DHA-based therapeutic approaches can significantly mitigate excitotoxic detrimental effects or improve neurological outcomes in humans.

Despite these favorable findings, the included studies revealed several limitations and inconsistencies. Many experimental models focused on early intervention after glutamate exposure, with limited investigation into chronic or age-related excitotoxicity. This is important, as aging populations are more susceptible to excitotoxins. Furthermore, the diversity of results among species and experimental models suggests that age, type of model, induction technique, and treatment settings may all affect the efficacy of DHA. While DHA showed protective benefits in seizure and epilepsy models, and improved cognitive function in FPI-induced TBI rats, its effect on motor recovery was inconsistent, indicating possible domain-specific effects. Another limitation is the inclusion of studies using mixed omega-3 or fish oil formulations. Although these studies specify the DHA dosage or concentration, the presence of EPA and other fatty acids complicates the determination of whether the observed beneficial effects were attributable solely to DHA or to the synergistic effect of other lipid components.

**Figure 5:**
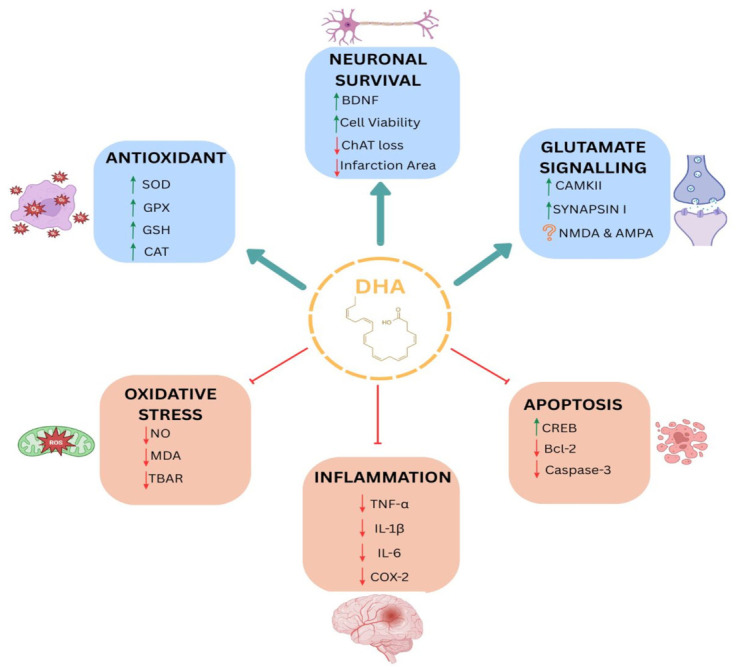
Proposed therapeutic effects of DHA in ameliorating glutamate-induced neurotoxicity.

Nonetheless, this comprehensive study elucidates the various neuroprotective mechanisms of DHA in excitotoxicity. This review focuses on the therapeutic roles of DHA in addressing oxidative stress, inflammation, and apoptosis in excitotoxicity from in vivo, ex vivo, and in vitro research. These findings may help guide future research towards optimizing experimental models and treatment options for excitotoxicity in neurological conditions. The proposed neuroprotective effects of DHA on glutamate-induced neurotoxicity are shown in Figure 5.

## 5. Conclusions

Taken together, DHA showed significant therapeutic effects against glutamate-induced neurotoxicity. Nonetheless, all the studies reviewed are preclinical investigations, with no randomized human clinical trials conducted to confirm the efficacy of DHA in neurological disorders. Therefore, further clinical investigations are recommended to verify the effects of DHA, assess its side effects, and examine its long-term use as a therapeutic candidate for neurological disorders.

## Figures and Tables

**Table 1:** Characteristics of selected in vivo studies on the neuroprotective effects of docosahexaenoic acid on glutamate-induced neurotoxicity.

In Vivo Study					
Study Design	Source of DHA	Treatment Dosage	Duration of Treatment	Findings	References
Young Wistar rats (14-day-old) from DHA-supplemented mothers and exposed to NMDA excitotoxin injection (40 nmol) in the Nucleus basalis of Meynert (NBM) for 48 h	Fish oil (Marinol D40)	16.7 g Marinol D40 per 100 g diet (containing DHA ~13.6% of total FA)	Maternal supplementation: 1 week pre-mating, continued throughout gestation and lactation period	↓ loss of cholinergic neuronal cell bodies in NBM. ↓ the loss of ChAT- and p75-positive neurons in NBM. ↓ the percentage loss of AChE fiber density at the superficial cortical layers I–IV. ↑ neuronal membrane DHA levels in all phospholipids.	[71]
Adult Sprague-Dawley rats were subjected to fluid percussion injury (FPI) to induce TBI	N/A	DHA-enriched diet (containing 1.2% DHA)	12 days post-FPI	↑ Learning ability in MWM. ↑ BDNF and ↑ CAMKII level in hippocampus. ↑ Syn-1 and ↑ CREB level in hippocampus. ↓ 4-HNE in hippocampus. ↑ SOD and ↑ Sir2 level in hippocampus. ↑ iPLA2 and ↑ STX-3 in hippocampus. ↑ DHA content in the cortical tissue.	[76]
Zebrafish offspring from PUFA-supplemented parents were treated with Kainate Acid (KA) injection (6 mg/kg BW)	N/A	200 mg/kg BW Dietary supplement administered at 1 month before spawning and fed to offspring until 20 months of age	Female zebrafish: 1 month prior to spawning Offspring: Until 20 months of age	↑ Survival rate in mother and offspring treated with DHA ↓ Epileptic scores (↓ seizure scores) in mother and offspring treated with DHA ↓ Seizure intensity in mother and offspring treated with DHA ↓ Statis epilepticus events in mother and offspring treated with DHA Better outcomes observed in both mother and offspring treated with DHA	[68]
Male offspring of Harlan-Wistar rats from DHA-supplemented mothers were induced with NMDA (180 nmol/brain), bilateral i.c. into the entorhinal cortex	Fish oil (Marinol d40) 1.67 g per 100 g of LC-PUFA-enriched diet (DHA comprises 13.6% of total fatty acids) (Hgyes et al., 2003 [71])	N/A Route of administration: Dietary supplementation	Female rats: one week pre-mating, continued throughout gestation and lactation until weaning	NS (*p* > 0.05) between escape latency and probe trial in the MWM between LC-PUFA and placebo groups ↓ neuronal lesion size by 40%. NS (*p* > 0.05) in microglial activation between LC-PUFA and placebo groups NS (*p* > 0.05) in size of microglial invasion inside and around the lesion	[70]
Male CDF rats were fed with fish oil supplemented diet received daily intraperitoneal injection of NMDA (25 mg/kg BW)	Fish oil (Zeigler Bros, Gardners, PA, USA) containing 5.1% α-LNA, 1.9% EPA and 2.4% DHA of total fatty acids (2.4 g/100 g)	N/A Route of Administration: Intraperitoneal Injection	19 weeks	NS (*p* > 0.05) in DHA, AA and n6-DPA composition in brain in fish-oil supplemented group ↓ cPLA2 level in fish-oil supplemented group NS (*p* > 0.05) in sPLA2 level compared to adequate diet group Fish oil supplementation maintains level of iPLA2 after chronic NMDA NS (*p* > 0.05) in Cox-1 and Cox-2 level compared to the adequate diet group NS (*p* > 0.05) in astrocytic, neurotrophic and inflammatory markers when comparing between fish oil supplementation and adequate diet group	[72]
Female CD-1 mice were injected with OGV 10% (5 mL/kg BW) after being induced with MCAO for 90 min and after reperfusion	Fish oil (Omegaven, Fresenius Kabi AG, Bad Homburg, Germany, OGV 10%^®^; DHA 18 mg/mL)	OGV 10%^®^: 5 mL/kg BW, i.v. (~90 mg/kg BW DHA), administered at 5 or 90 min post-MCAO (stroke onset vs. reperfusion) ROD: Injection	24 h	**Treatment at Reperfusion (a.r.):** ↓ Infarct area in the brain slice by 21% and stroke severity by 50% Restored mitochondrial function in brain tissues (↑ MMP by 20%, ↑ ATP by 63%, ↑ RCR by 31%) **Treatment at stroke onset (a.s.):** ↓ Infarct area in the brain slice by 56% and stroke severity by 60% ↑ Motor function through the observation of neurobehavioral assessment ↓ Glutamate levels in the ischemic striatum region during stroke ↑ Glucose levels in the ischemic striatum region after reperfusion ↓ Brain IL-6 and ↓ COX-2 levels NS (*p* > 0.05) in brain IL-10 levels	[73]
Female wistar-albino rats were fed with MSG (4 mg/g/day BW) together with DHA (300 mg/kg BW) for 9 days	N/A	300 mg/kg BW of DHA being fed together with 4 mg/g MSG BW Route of Administration: Oral gavage	9 days	↑ NMDAR, ↑ BDNF and ↑ NPY in CA1 and DG region of the DHA and EPA treated rats NS (*p* > 0.05) between DHA and EPA treated groups through ELISA and IHC of the hippocampal region	[75]
Male offspring of Wistar rats from DHA-supplemented mothers were induced with MSG (2 g/kg BW) for 10 alternate days	Fish oil (Mega™-3) Microalgal biomass (*Isochrysis* sp.)	Microalgal biomass: 1.4 mg/kg BW/day, p.o. (post-MSG induction) Fish oil: 2.9 mg/kg BW/day, p.o. (post-MSG induction)	Female rats: 30 days pre-mating and continued throughout gestation and lactation Male offspring of Wistar rats: postnatal treatment for 30 days	↑ Body weight in the Microalgal group (MG) and Fish-oil group (FG) ↓ Brain MDA levels in both MG and FG NS (*p* > 0.05) in brain protein carbonyl levels in MG and FG ↓ NMDA2b gene expression levels in the brain of MG and FG ↑ BDNF and CREB in the brain of MG and FG ↓ Bcl-2 and caspase-3 gene expression levels in brain of MG and FG Antioxidant activities in the brain: - MG: ↓ SOD, ↓ CAT, ↑ GPX levels - FG: ↑ SOD, ↑ CAT, ↑ GPX levels ↓ Neuronal damage in the cerebrum of FG and MG ↑ Brain DHA and ARA levels	[69]
Adult Wistar rats were fed with omega-3 PUFA diet and MSG (4 mg/g/day BW) for four weeks	1000 mg fish oil capsule (Natrol^®^ Omega-3 fish oil)	48 mg of DHA for every 400 mg/kg BW ω−3 PUFA diet being fed together with 4 mg/g BW of MSG Route of Administration: Gastric gavage	4 weeks	**Redox State in the brain cortex:** ↓ MDA, ↓ GSH and ↓ SOD ↑ CAT NS (*p* > 0.05) in NO level **Neuroinflammation in the brain cortex:** ↓ TNF-a and ↓ IL-1B **Neurochemical parameters:** ↓ MAO and ↓ AChE activity ↑ 5-HT and ↑ DA level **Apoptotic Markers:** ↓ caspase-3 production ↓ Swelling, ↓ apoptosis ↓ inflammation, ↓ nerve cell damage and ↓ vascular congestion through IHC staining of the cerebral cortex	[74]

Note: Upward arrow (↑) indicate increased; Downward arrow (↓) indicate decreased; Not significant (NS); *N*-methy-D-aspartate (NMDA); α-amino-3-hydroxy-5-methyl-4-isoxazolepropionic acid (AMPA) Excitatory Amino Acid Transporters(EAAT); Long-chain Polyunsaturated Fatty Acid (LC-PUFA); Docosahexaenoic Acid (DHA); Fluid Percussion Injury(FPI); Nucleus Basalis of Meynert (NBM); Kainate Acid (KA); Body Weight (BW); Monosodium Glutamate (MSG); Interleukin 1 Beta (IL-1β); Tumor Necrosis Factor Alpa (TNF-α); Eicosapentaenoic Acid (EPA); Calcium/calmodulin-dependent protein kinase II (CAMKII); Glutathione (GSH); Lactate Dehydrogenase (LDH); Cornu Ammonis 1 (CA1); Dentate gyrus (DG); Mitochondria Membrane Potential (MMP); Respiratory Control Ratio (RCR); Superoxide dismutase (SOD); Glutathione Peroxidase (GPX); Catalase (CAT), Arachidonic acid (ARA).

**Table 2:** Characteristics of selected in vitro and ex vivo studies on the neuroprotective effects of docosahexaenoic acid on glutamate-induced neurotoxicity.

In Vitro/Ex Vivo Study					
Study Design	Source of DHA	Treatment Dosage	Duration of Treatment	Findings	References
Hippocampus slices from SD rat pups were induced with 0.5 mM L-glutamate for 1 h	DHA (Sigma, St. Louis, MO, USA)	The hippocampal slices were pre-treated with 5 μg/mL, 30 μg/mL or 50 μg/mL of DHA either for 24 h or 30 min before being induced with 0.5 mM L-glutamate	Pre-incubation 0.5 h or 24 h before L-glutamate exposure	↑ Cell viability. ↑ GSH level. ↑ GSH-Px and ↑ GR. ↓ NO production. ↓ Calcium ion entry.	[78]
Hippocampus slices from SD rats induced with glutamate receptor agonists (10 µM AMPA, 10 µM NMDA, 20 µM KA) for 24 h	DHA (Ann Arbor, MI, USA)	Hippocampal slices from 12-day-old rats were pre-incubated with 90 μM or 30 μM of DHA solution were for 24 h before NMDA (10 µM), AMPA (10 µM) and KA (20 µM) treatment	Pre-incubation for 24 h	↓ LDH in AMPA-induced toxicity with DHA pre-treatment but NS (*p* > 0.05) in NMDA or KA-induced toxicity ↓ PI uptake (cell death marker) in CA1 region observed in DHA treated group ↓ GluR1 and GluR2 protein level with DHA NS (*p* > 0.05) in NR1 and NR2A protein expression with DHA treatment	[77]
Primary oligodendrocytes from mixed glial cultures of P1 mouse brain were induced with 25 μM AMPA for 5 min	DHA (Sigma, St. Louis, MO, USA)	Primary oligodendrocytes were pre-treated with 20 μM DHA for 48 h before being exposed to AMPA (25 μM) for 5 min	Pre-incubation for 48 h	↓ LDH level in AMPA+DHA treated oligodendrocytes ↓ AMPA-induced oligodendrocyte cell death with DHA pre-treatment ↓ Degeneration of oligodendrocyte during DHA-pre-treated + LPS microglia and oligodendrocyte co-culture DHA downregulates the activation of microglia during co-culturing with DHA-treated oligodendrocytes and protect against oligodendrocyte apoptosis	[79]
Hippocampus slices from male Swiss mice were induced with 10 mM glutamate for 2 h	DHA (Sigma, St. Louis, MO, USA)	Hippocampal slices were pre-incubated with 5 μM of DHA for 15 min before the treatment of 10 mM glutamate	Pre-incubation for 15 min	↑ Cell viability when being pre-incubate with DHA ↑ Cell viability after treatment with glutamate in DHA pre-incubated group NS (*p* > 0.05) in glutamate uptake compared to the control group ↓ TBAR level in DHA-treated group PPADS (P2 receptor antagonist) inhibits the neuroprotective effects of DHA Activation of adenosine happens with DHA treatment DHA pre-incubation of hippocampal slices protects from cellular damage at high concentrations of glutamate exposure, while not affecting the amount of glutamate uptake during excitotoxicity	[56]

Note: Upward arrow (↑) indicate increased; Downward arrow (↓) indicate decreased; Not significant (NS); Nitric Oxide (NO); Cornu Ammonis 1 (CA1); Glutathione (GSH); Lactate Dehydrogenase (LDH); *N*-methy-D-aspartate (NMDA); α-amino-3-hydroxy-5-methyl-4-isoxazolepropionic acid (AMPA); Kainate Acid (KA); Lipoposaccharide (LPS); Thiobarbituric Acid Reactive Substances (TBAR); Pyridoxal phosphate-6-azophenyl-2′,4′-disulfonic acid (PPADS).

## Data Availability

No new data were created or analyzed in this study. Data sharing is not applicable to this article.

## Abbreviations

The following abbreviations are used in this manuscript:

CNS	Central Nervous System
NMDA	*N*-methy-D-aspartate
AMPA	α-amino-3-hydroxy-5-methyl-4-isoxazolepropionic acid
EAAT	Excitatory Amino Acid Transporters
AD	Alzheimer’s Disease
PD	Parkinson’s Disease
TBI	Traumatic Brain Injury
LC-PUFA	Long-chain Polyunsaturated Fatty Acid
DHA	Docosahexaenoic Acid
PC	Phosphatidylcholine
TAG	Triacylglycerol
SYRCLE	Systematic Review Centre for Laboratory Animal Experimentation
JBI	Joanna Briggs Institute
FPI	Fluid Percussion Injury
NBM	Nucleus Basalis of Meynert
KA	Kainate Acid
BW	Body Weight
MSG	Monosodium Glutamate
SPM	Pro-resolving Mediators
NPD1	Neuroprotectin D1
IL-1β	Interleukin 1 Beta
TNF-α	Tumor Necrosis Factor Alpa
EPA	Eicosapentaenoic Acid
CAMKII	Calcium/calmodulin-dependent protein kinase II
NO	Nitric Oxide
GSH	Glutathione
LDH	Lactate Dehydrogenase
NS	Not Significant
